# Contrast Agent Uptake in Endolymphatic Sac and Duct: Inverse Relation to Endolymphatic Hydrops

**DOI:** 10.1002/lary.32127

**Published:** 2025-03-19

**Authors:** Johannes Gerb, Emilie Kierig, Valerie Kirsch, Sandra Becker‐Bense, Rainer Boegle, Thomas Brandt, Marianne Dieterich

**Affiliations:** ^1^ German Center for Vertigo and Balance Disorders LMU University Hospital, LMU Munich Munich Germany; ^2^ Department of Neurology LMU University Hospital, LMU Munich Munich Germany; ^3^ Graduate School of Systemic Neuroscience LMU Munich Munich Germany; ^4^ Munich Cluster for Systems Neurology (SyNergy) Munich Germany

**Keywords:** endolymphatic duct, endolymphatic hydrops, endolymphatic sac, inner ear, Ménière's disease, vertigo, vestibular migraine, vestibulopathy

## Abstract

**Objectives:**

Ménière's disease (MD) and vestibular migraine (VM) can be associated with endolymphatic hydrops (ELH). The differential role of the endolymphatic sac and duct (ES/ED) system for the development of ELH is poorly understood.

**Methods:**

On 251 delayed, contrast‐enhanced inner ear MRI (iMRI) datasets from neurotological patients and healthy control participants, we evaluated (1) the visibility of the ES/ED system using a novel semi‐quantitative scale, and (2) the dimensions of ELH, calculated using volumetric local thresholding (VOLT). Afterwards, statistical analysis of ES/ED radiologic visibility in relation to the grade of ELH, the degree of clinical symptoms, and audiometric findings was performed.

**Results:**

Patients were divided into an MD cohort (*n* = 68, 34 females, mean age 54.5 ± 14.8 years) and a VM cohort (*n* = 67, 42 females, 45.9 ± 15.5 years). The remaining datasets did not fulfill diagnostic criteria for definite diagnoses (*n* = 64, 27 females, mean age 51.3 ± 16.6) or were from healthy controls (HC; *n* = 52, 27 females, 49.0 ± 18.1 years). MD patients showed the lowest ES/ED‐visibility scores on the affected side (ANOVA F(172,2): 20.60, *p* < 0.001), while the ES/ED‐visibility on the non‐affected side in MD patients was still significantly lower than in VM and HC (ANOVA F(172,2): 6.80, p 1.44 × 10^−3^). The ES/ED‐visibility score and ELH volume (determined by VOLT, in mm^3^) correlated inversely (Spearman's rho: −0.32, Fisher's *z* −0.34, *p* < 0.001).

**Conclusion:**

ES/ED radiologic visibility in iMRI is inversely associated with ELH volumes. Patients with MD show substantially decreased ES/ED visibility on the affected ear and (less pronounced) on the unaffected ear, while VM and HC exhibit normal ES/ED visibility.

**Level of Evidence: 3.:**

## Graphical abstract

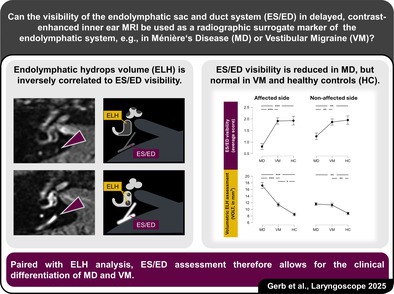

Question: Is there a disease‐specific abnormality of the endolymphatic sac and duct system (ES/ED) in delayed, contrast‐enhanced inner ear MRI in patients with Ménière's disease (MD) and vestibular migraine (VM) that correlates with the endolymphatic hydrops (ELH)?

Findings: In a cohort study of 251 adults, an inverse correlation was disclosed between ES/ED visibility and ELH Volume with a decreased ES/ED visibility on both (affected and unaffected) sides in MD, as distinct from VM.

Meaning: The use of delayed post‐contrast MRI provides a radiographic marker for the differentiation of MD and VM, compatible with a reduced inner ear endolymph resorption.

## 1 Introduction

Imaging of the inner ear fluid compartments by magnetic resonance tomography is a valuable research tool, which can aid in the diagnosis of vestibular disorders [1, 2]. Intravenous admission of contrast agent (CA) after 4–5 h causes a CA enrichment within the perilymphatic fluid in contrast to the non‐enriching endolymphatic fluid. This contrast allows the visualization and quantification of endolymphatic hydrops (ELH), a common finding in Ménière's disease (MD) [3, 4]. However, ELH also occurs in other vestibular syndromes such as vestibular migraine (VM) [5, 6, 7], and might constitute a disease‐related epiphenomenon rather than play a direct causative role in MD [8]. The degree of ELH can be graded semi‐quantitatively or volumetrically [3, 9, 10].

Recently, the use of endolymphatic sac (ES) or duct (ED) imaging parameters in delayed contrast‐enhanced MRI of the inner ear has been shown to allow clinical endotyping of MD, with many MD patients exhibiting a reduced CA uptake within these structures in 4 h delayed inner ear MRI [11, 12, 13]. The endotypes defined by these radiographic surrogate markers (such as the angular trajectory of the vestibular aqueduct) often correlate with specific phenotypic features [14].

This finding is in line with histopathological studies showing distinct MD subtypes [15]. Physiologically, the ES/ED system plays an important role in endolymph homeostasis [16], but also in the inner ear immune response [17]. Impaired ES/ED function with reduced endolymphatic fluid resorption has therefore been postulated as a key factor for the development of ELH [18]. However, a quantitative proof of a direct relationship between in vivo ELH volume and ES/ED system integrity by imaging parameters is still lacking.

In the current study, we therefore aimed to (i) quantitatively analyze delayed post‐contrast MRI visibility of the ES/ED system in patients with neurotological disorders such as MD and VM; (ii) investigate the relationship between ELH and ES/ED visibility; and (iii) correlate the inner ear MRI findings with clinical data.

## 2 Materials and Methods

### 2.1 Setting and Institutional Review Board Approval

All data was acquired at the Interdisciplinary German Center for Vertigo and Balance Disorders at Munich University Hospital (LMU Munich), Germany, between 2015 and 2020. Approval of the institutional review board of Ludwig‐Maximilians university was obtained before the initiation of the study (no. 641‐15). All participants provided informed oral and written consent in accordance with the Declaration of Helsinki and its later amendments before inclusion in the study.

### 2.2 Study Population and Clinical Testing

Inner ear datasets (*n* = 502) from 251 patients who underwent iMRI for exclusion or verification of ELH were included. Exclusion criteria were neurological or psychiatric disorders, as well as any MR‐related contraindications [19], poor image quality, or missing MR sequences. The diagnostic workup consisted of a thorough neurological and audiological workup (e.g., history‐taking and clinical examination), a detailed neuro‐orthoptic assessment (e.g., Frenzel glasses, fundus photography and adjustments of the subjective visual vertical), as well as neurotological testing (video‐oculography during bithermal caloric irrigation and head impulse testing, vestibular evoked myogenic potentials, pure tone audiometry). Clinical diagnosis of vestibular disorders was based on international diagnostic criteria by the Bárány Society. In their current version, these guidelines do not require ELH imaging for diagnosis; all patients were aware that ELH imaging was performed for research purposes. Patients filled out the Dizziness Handicap Inventory (DHI [20]), the Vertigo Symptom Scale (VSS [21]), the Migraine Disability Assessment (MIDAS [22]) and the Headache Impact Test (HIT‐6 [23]).

### 2.3 Delayed Intravenous Gadolinium‐Enhanced MRI of the Inner Ear

Four hours after intravenous injection of a standard dose (i.e., 0.1 mmol/kg body weight) of gadobutrol (Gadovist, Bayer, Leverkusen, Germany), subjects underwent MR imaging in a whole‐body 3 Tesla MRI scanner (Magnetom Skyra, Siemens Healthcare, Erlangen, Germany) with a 20‐channel head coil. For assessing ES/ED visibility, a T2‐weighted, three‐dimensional, fluid‐attenuated inversion recovery sequence (3D‐FLAIR) was acquired (TR 6000 ms, TE 134 ms, TI 2240 ms, FOV 160 × 160 mm^2^, 36 slices, base resolution 320, averages 1, acceleration factor of 2 using a parallel imaging technique with a generalized auto‐calibrating partially parallel acquisition (GRAPPA) algorithm, slice thickness 0.5 mm, acquisition time 15:08 min). For inner ear cisternography, a high‐resolution, strongly T2‐weighted, 3D constructive interference steady state (CISS) sequence of the temporal bones was performed (TR 1000 ms, TE 133 ms, FA 100°, FOV 192 × 192 mm^2^, 56 slices, base resolution 384, averages 4, acceleration factor of 2 using GRAPPA algorithm, slice thickness of 0.5 mm and acquisition time 8:36 min).

### 2.4 ES/ED Visibility Scale

**FIGURE 1:**
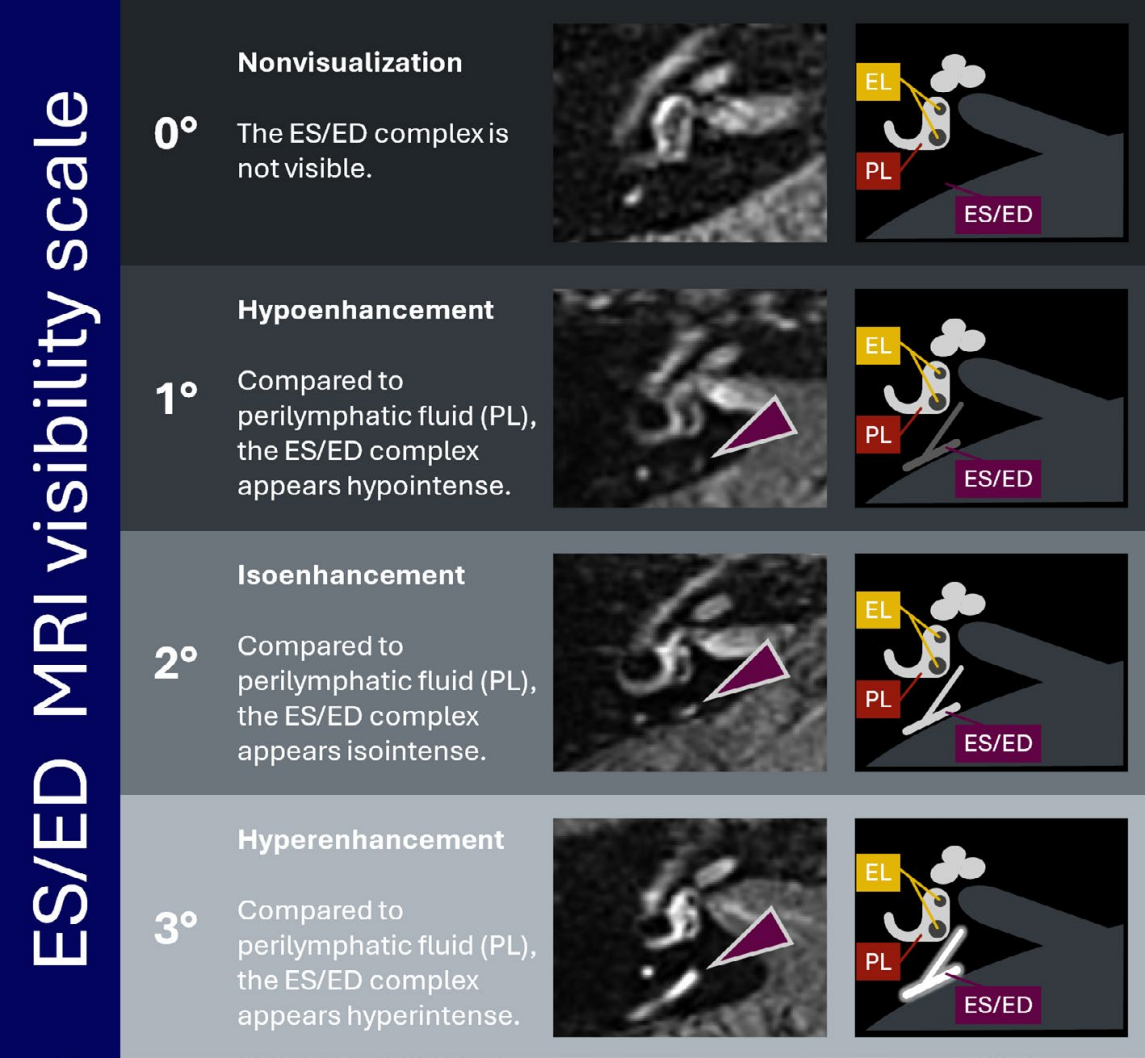
ES/ED visibility scale for 4 h post‐contrast 3D‐FLAIR images. At midmodiolar level on an axial slice of the inner ear, the ES/ED complex (marked in purple arrow) is identified and schematically depicted (right column). ES/ED image intensity is then compared to PL (marked in dark red) intensity: ES/ED 0° stands for no visibility (nonvisualization), ES/ED grade 1° stands for lower intensity (hypoenhancement), ES/ED grade 2° stands for similar intensity (isoenhancement), and ES/ED 3° stands for higher intensity (hyperenhancement). Note that the grade of endolymphatic hydrops, i.e., a potential increase of EL (marked in yellow), is not part of the ES/ED‐scale. [Color figure can be viewed in the online issue, which is available at www.laryngoscope.com.]

In order to quantify the visibility of the ES/ED system, we used a novel semi‐quantitative 4‐point rating scale (Figure 1). For grading, an axial slice from the 3D‐FLAIR depicting the inner ear at midmodiolar height was selected, and the ED was followed in a caudal direction until the ES was identified. In this grading system, 0° stands for no visibility of the ES/ED complex (non‐visualization). If the ES/ED system can be visually identified, its intensity is then compared to the PLS intensity in the vestibulum at midmodiolar level: in grade 1°, the ES/ED intensity is lower (hypoenhancement); in grade 2° it is similar (isoenhancement), and in grade 3° it is higher (hyperenhancement, see Figure 1).

### 2.5 ELH Assessment

Using a previously described semi‐automatic ELH analysis pipeline (VOLT [10]), ELH volumes were calculated for each dataset. This method allows reproducible and objective volumetric ELH assessment and utilizes a combination of deep learning segmentation and three‐dimensional local thresholding image analysis to calculate the amount of ELH fluid in mm^3^. Additionally, clinical grading using the semi‐quantitative scale from Baráth et al. [3] was performed by experienced neuroradiologists. In this grading system, ELH characteristics are assessed visually, and a set of image‐based criteria is used to rate the degree of ELH.

### 2.6 Statistical Analysis

For further statistical analysis, JASP 0.18.3 (jasp‐stats.org) was used. Correlation between continuous variables was assessed using Spearman's rho, while binomial group comparisons were conducted using independent samples *t*‐tests, and group comparisons for more than two groups were conducted using ANOVA with Tukey correction. *P*‐values were controlled for multiple testing. In patients without a clinically leading ear (i.e., VM and HC cohort), a pseudorandom number generator [6] was used to assign “affected” and “non‐affected” sides for cohort analyzes.

## 3 Results

Of the 251 patients enrolled (mean age 50.3 ± 16.4 years, 130 females), the MD cohort consisted of 68 (34 females, mean age 54.5 ± 14.8), the VM cohort of 67 (42 females, mean age 45.9 ± 15.5), and the “vestibular healthy” controls of 52 patients (27 females, mean age 49.0 ± 18.1). The remaining datasets of 64 patients (27 females, mean age 51.3 ± 16.6) who did not fulfill the international diagnostic criteria for *definite* diagnoses, but only probable VM or MD, remained part of the general correlation analysis of ELH volume and ES/ED visibility but were excluded from the analysis of disease‐associated effects.

### 3.1 Clinical Details of MD and VM Cohort

The MD cohort had a higher mean age than the VM cohort (see Table 1 for details). No significant differences in disease duration were found. In vestibuloauditory testing, the MD cohort exhibited deficits on the affected side, while both groups had comparable findings on the non‐affected side (affected and non‐affected side were randomly assigned in the VM cohort, as described above). In the psychometric tests, the VM cohort typically described higher disease impact.

**TABLE 1:** Clinical findings in the MD and VM cohort.

	Unit	MD	VM	Welch's *t*‐test
N (of which females)	—	68 (34)	67 (42)	—
Age	Years	54.5 ± 14.8	45.9 ± 15.5	*t* 3.31, *p* 1.20 ×10^−3^, Cohen's d 0.57
Disease duration	Months	56.4 ± 59.1	69.0 ± 114.0	n. s.
Ipsilateral mean caloric excitability	°/s	8.24 ± 7.51	11.31 ± 5.70	*t* −2.18, *p* 0.03, Cohen's d − 0.46
Contralateral mean caloric excitability	°/s	12.42 ± 8.60	11.93 ± 7.88	n. s.
Ipsilateral vHIT gain	—	0.82 ± 0.21	1.01 ± 0.30	*t* −3.07, *p* 3.73 ×10^−3^, Cohen's d − 0.74
Contralateral vHIT gain	—	0.86 ± 0.16	0.89 ± 0.15	n. s.
Ipsilateral low‐frequency hearing loss	dB	43.26 ± 26.49	18.81 ± 14.64	*t* 5.80, *p* < 0.001, Cohen's d 1.14
Contralateral low‐frequency hearing loss	dB	22.61 ± 16.59	16.81 ± 10.87	n. s.
MIDAS	Average grade (1: no migraine‐related disability, 4: maximum disability)	2.62 ± 1.37	2.97 ± 1.24	n. s.
HIT‐6	Average grade (1: no impact, 4: maximum impact of headaches on daily functioning)	1.90 ± 1.17	2.33 ± 0.99	*t* −2.31, *p* 0.03, Cohen's d − 0.40
DHI	Points	34.88 ± 19.95	43.08 ± 22.00	*t* −2.20, *p* 0.03, Cohen's d − 0.39
VSS	Points	29.50 ± 17.56	37.19 ± 21.37	*t* −2.13, *p* 0.04, Cohen's d − 0.39

*Note*: Ipsilateral refers to the clinically affected side, and contralateral refers to the unaffected side. In VM patients, sides were assigned using a pseudorandom number generator. Note that the Migraine Disability Assessment (MIDAS) focuses on headache‐associated disability, which often did not adequately represent VM patient symptomatology.

Abbreviations: DHI, Dizziness handicap inventory; HIT‐6, headache impact test; MIDAS, Migraine disability assessment; VSS, Vertigo symptom scale; vHIT, video head impulse test.

All MD patients were recommended betahistine treatment according to internal guidelines (recommended daily dosage: 72 mg). In case of symptom progression, some patients were treated with a higher daily dosage of up to 216 mg. At the time of MRI acquisition, 15 patients did not take any betahistine, 26 took lower dosages than recommended, 16 took the recommended dosage, and 5 took higher dosages. From the remaining six patients, no data was available. Betahistine dosage showed no effect on ipsi‐ or contralateral ES/ED visibility, ipsi‐ or contralateral semiquantitative ELH grading, or ipsi‐ or contralateral volumetric (VOLT) ELH analysis (ANOVA: n. s.). None of the patients were undergoing diuretic treatment at the time of MRI acquisition, and none had undergone prior endolymphatic shunt surgery.

### 3.2 ES/ED Visibility, ELH Volume and ELH Grading

**FIGURE 2:**
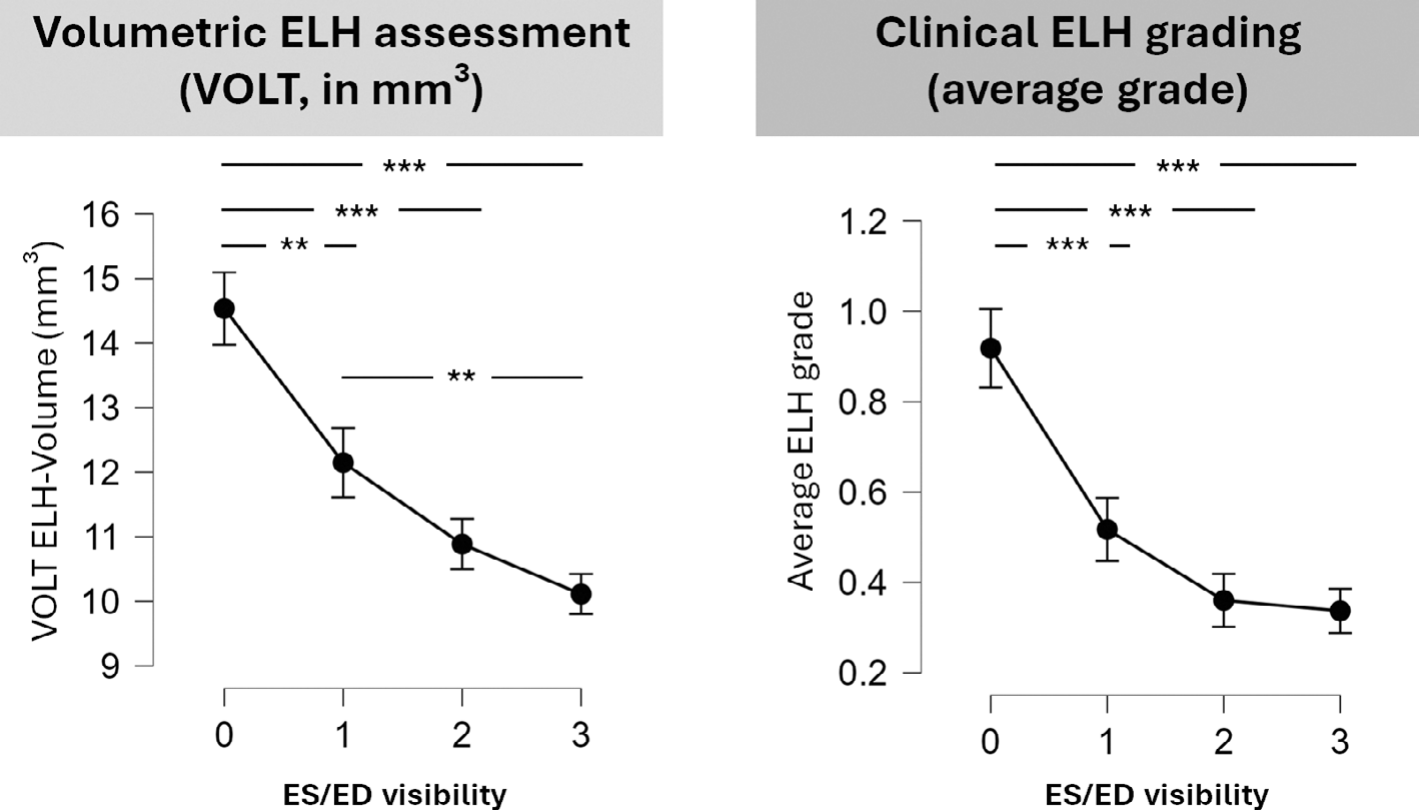
Plots of ES/ED visibility and quantitative volumetric ELH assessment (volume in mm^3^, left) and clinical visual ELH grading (right) from 251 delayed contrast‐enhanced inner ear datasets (502 individual inner ears). Lower ES/ED visibility exhibits a direct association with higher ELH volumes (calculated using VOLT), and with higher clinical ELH gradings (* = *p* < 0.05, ** = *p* < 0.01, *** = *p* < 0.001, Tukey‐corrected ANOVA).

In all inner ear datasets (*n* = 502), i.e., without any a priori filtering of diagnoses, ES/ED visibility score and ipsilateral ELH volume (determined by VOLT, i.e., an algorithm‐based, volumetric volume calculation of inner ear fluid compartments) were inversely correlated: higher ELH volumes were associated with lower ES/ED visibility, and vice versa (Spearman's rho side: −0.32***, Fisher's z −0.34, *p* < 0.001). ES/ED visibility score and clinical semi‐quantitative ELH grading were also inversely correlated (Spearman's rho: −0.21***, Fisher's *z* −0.21, *p* < 0.001). As expected, the VOLT volumes correlated positively with clinical grading (Spearman's rho: −0.42***, Fisher's *z* −0.45, *p* < 0.001). ANOVA testing revealed significant differences after grouping by ES/ED visibility both for the ELH volumes (F(492,3) = 20.34, *p* < 0.001, mean ELH volume ES/ED 0*°*: 14.54 ± 6.36, ES/ED 1*°*: 12.15 ± 5.44, ES/ED 2*°*: 10.89 ± 3.95, ES/ED 3*°*: 10.11 ± 3.95) and for the clinical gradings (F(334,3) = 16.71, *p* < 0.001, mean ELH grade ES/ED 0*°*: 0.92 ± 0.84, ES/ED 1*°*: 0.52 ± 0.60, ES/ED 2*°*: 0.36 ± 0.47, ES/ED 3*°*: 0.34 ± 0.51; see Figure 2).

Independent of the clinical diagnosis, higher patient age correlated with lower ES/ED visibility (Spearman's rho −0.21***, Fisher's *z* −0.21, *p* < 0.001). Higher age additionally correlated with higher ELH volume determined by VOLT (Spearman's rho 0.10*, Fisher's *z* 0.10, *p* < 0.05), but not with the semi‐quantitative visual grading (Spearman's rho n. s.). When analyzing individual patient datasets independent of diagnosis, ES/ED visibility score and ipsilateral ELH volume correlated inversely on both sides (Spearman's rho left side: −0.38***, Fisher's *z* −0.40, *p* < 0.001; right side: −0.24***, Fisher's *z* −0.25, *p* < 0.001). ES/ED visibility and contralateral ELH volume correlated as well, albeit with a smaller effect size (Spearman's rho left side: −0.26***, Fisher's *z* −0.27, *p* < 0.001; right side: −0.16*, Fisher's *z* −0.16, *p* < 0.05). ES/ED visibility score and ipsilateral clinical semi‐quantitative ELH grading correlated on both ears (Spearman's rho left side: −0.16*, Fisher's *z* −0.16, *p* < 0.05; right side: −0.20**, Fisher's *z* −0.21, *p* < 0.01). A correlation with the contralateral side could be observed (Spearman's rho left side: −0.17**, Fisher's *z* −0.18, *p* < 0.01; right side: −0.14*, Fisher's *z* −0.15, *p* < 0.05). The VOLT volumes correlated well with the ipsilateral clinical grading (Spearman's rho left side: −0.29***, Fisher's *z* −0.30, *p* < 0.001; right side: −0.33***, Fisher's *z* −0.34, *p* < 0.001), and were not affected by contralateral ELH (Spearman's rho with contralateral side: n. s.).

Patients with unilateral tinnitus showed ipsilaterally reduced ES/ED visibility (Welch's *t*‐test ipsilateral ES/ED visibility: *t* = 1.83, *p* 0.03*, contralateral ES/ED visibility: *t* = 1.26, *p* = 0.11), while patients with bilateral tinnitus had higher ES/ED visibility on both sides (Welch's *t*‐test ipsilateral ES/ED visibility: *t* = −2.11, *p* = 0.02*, contralateral ES/ED visibility: *t* = −1.97, *p* = 0.03*). Patients with unilateral aural fullness showed reduced ES/ED visibility on both sides (Welch's *t*‐test ipsilateral ES/ED visibility: *t* = 2.90, *p* 2.31 × 10^−3^**, contralateral ES/ED visibility: *t* = 2.38, *p* 9.69 × 10^−3^**), while patients with bilateral aural fullness exhibited a higher ES/ED visibility on both sides (Welch's *t*‐test ipsilateral ES/ED visibility: *t* = −1.47, *p* = 0.08, contralateral ES/ED visibility: *t* = −1.31, *p* = 0.10). Migraine symptoms were typically associated with bilaterally high ES/ED visibility, e.g., for headache (Welch's *t*‐test ipsilateral ES/ED visibility: *t* = −4.01, *p* = 5.04 × 10^−5^***, contralateral ES/ED visibility: *t* = −2.73, *p* = 3.57 × 10^−3^**), and phono/photophobia: (Welch's *t*‐test ipsilateral ES/ED visibility: *t* = −1.46, *p* = 0.07, contralateral ES/ED visibility: *t* = −1.64, *p* = 0.05*). No linear correlation between ES/ED visibility and respective ipsilateral low‐ and high‐frequency peripheral‐vestibular function was observable (ES/ED and ipsilateral caloric excitability: n. s.; ES/ED visibility and ipsilateral vHIT gain: n. s.), but ES/ED visibility showed a highly significant negative correlation with ipsilateral low‐frequency hearing impairment (Spearman's rho −0.27, *p* 5.33 × 10^−3^, Fisher's *z* −0.28). No linear association between ES/ED visibility and the psychometric tests (DHI, VSS, MIDAS, HIT‐6) was found.

### 3.3 Disease‐Related Effects

In patients with MD, “affected” refers to the clinically leading ear, and “non‐affected” refers to the opposite ear. In patients presenting without a leading clinical side and in the HC cohort, a pseudorandom number generator was used to assign “affected” and “non‐affected” sides.

**FIGURE 3:**
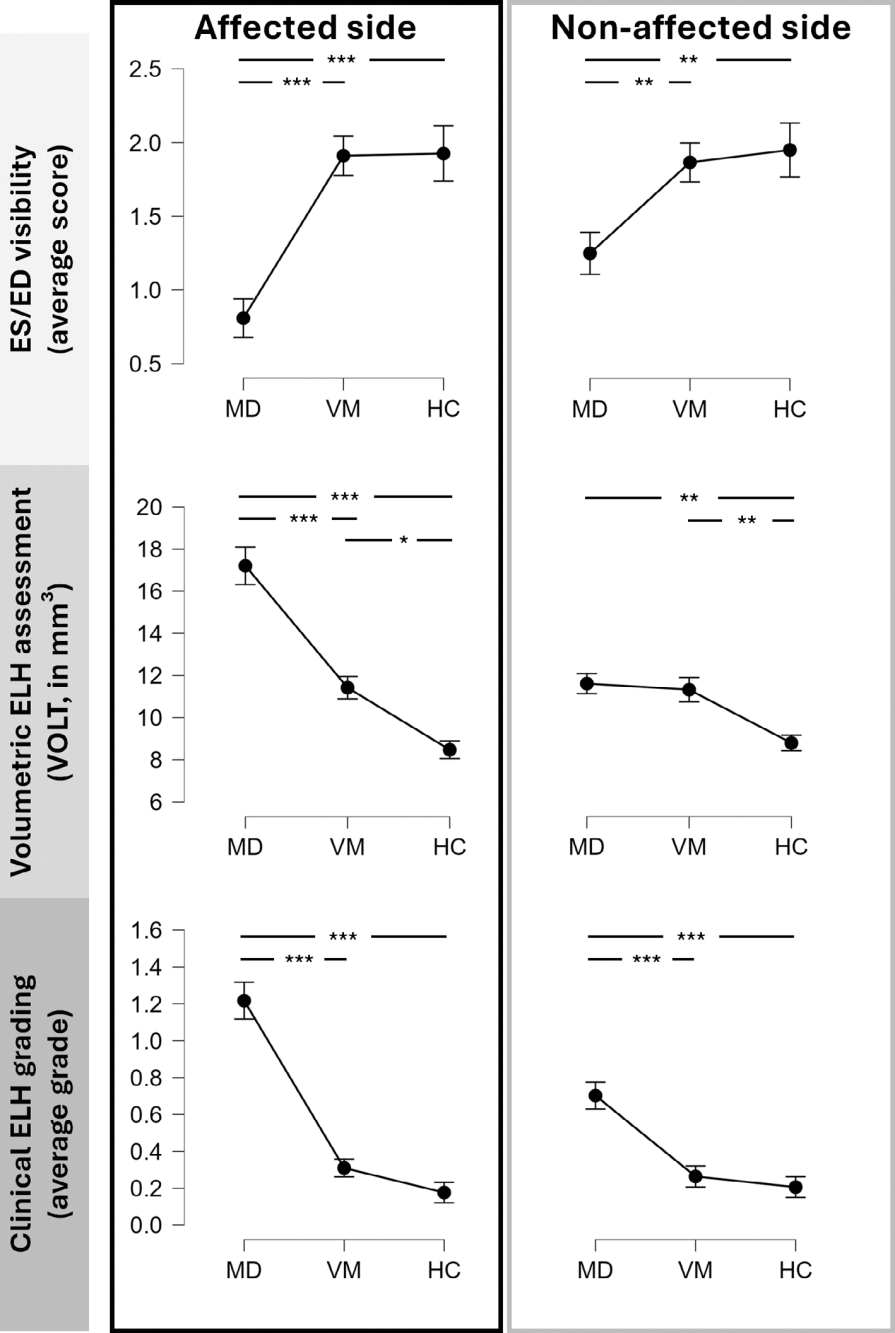
Plots of ES/ED visibility, ELH volumes determined by VOLT^6^, and clinical visual ELH grading for affected (red rectangle) and non‐affected patient side (blue rectangle), grouped for MD (*n* = 68), VM (*n* = 67) and vestibular healthy controls (HC, *n* = 48). For VM without clinically leading sides and HC, assignment of affected and non‐affected side was performed using a pseudorandom number generator. On the affected side, MD patients presented with the lowest average ES/ED visibility, the highest ELH volume, and the highest clinical ELH grading. On the non‐affected side, MD patients showed typically slightly higher ES/ED visibility, lower ELH volumes, and lower ELH gradings. VM and HC presented with good ES/ED visibility, and the VM cohort exhibited mild, bilateral ELH only detectable using VOLT (* = *p* < 0.05, ** = *p* < 0.01, *** = *p* < 0.001, Tukey‐corrected ANOVA).

The ES/ED‐score correlated well with the clinical diagnosis: MD patients showed the lowest scores on the affected side (ANOVA F(172,2): 20.60, *p* < 0.001, mean score MD: 0.81 ± 1.08; VM: 1.91 ± 1.10; HC: 1.93 ± 1.19), while the ES/ED‐visibility on the non‐affected side in MD patients was still significantly lower than in VM and HC (ANOVA F(172,2): 6.80, *p* 1.44 × 10^−3^, mean score MD: 1.25 ± 1.18; VM: 1.87 ± 1.09; HC: 1.95 ± 1.15). The ELH volumes (calculated using VOLT) were the highest on the affected side in MD (ANOVA F(164,2): 34.37, *p* < 0.001, mean score MD: 17.20 ± 7.23; VM: 11.42 ± 4.34; HC: 8.47 ± 2.44), while ELH volumes were comparable to VM (but still higher than HC) on the non‐affected side (ANOVA F(164,2): 6.18, p 2.58 × 10^−3^, mean score MD: 11.61 ± 3.87; VM: 11.32 ± 4.73; HC: 8.79 ± 2.17). For the clinical ELH gradings, again, MD showed the highest ratings on the affected side (ANOVA F(166,2): 52.00, *p* < 0.001, mean score MD: 1.22 ± 0.83; VM: 0.31 ± 0.39; HC: 0.18 ± 0.32) while a slight hydrops was seen on the non‐affected side (ANOVA F(166,2): 16.86, *p* < 0.001, mean score MD: 0.70 ± 0.60; VM: 0.26 ± 0.47; HC: 0.21 ± 0.33, Figure 3).

## 4 Discussion

In this study, a direct inverse relationship between ES/ED visibility and ELH severity in delayed, contrast‐enhanced inner ear MRI could be demonstrated: the more pronounced the ELH, the lower the ES/ED contrast agent uptake. ES/ED visibility was asymmetrically decreased in MD patients, with the clinically affected side exhibiting the lowest visibility. ES/ED visibility was bilaterally high in VM and HC participants. Independent of the clinical diagnosis, higher patient age was associated with lower ES/ED visibility and higher ELH volumes. Lower ES/ED visibility was associated with MD‐typical clinical signs and showed a strong correlation with low‐frequency hearing deficits.

This approach, i.e., comparing detailed volumetric ELH analyzes with ES/ED post‐contrast visibility analyzes, allows for an in vivo assessment of the endolymphatic fluid system. Potentially, impairment of this fluid system, measured e.g., by analyzing post‐contrast ES/ED visibility, could be used as a diagnostic radiographic marker in vestibular disorders. Earlier studies on the etiology and development of ELH proposed both hypersecretion of endolymphatic fluid and decreased resorption as pathophysiologically relevant factors. However, the microanatomy of the ES/ED complex and the in vivo fluid dynamics are only poorly understood [24]. While peri‐endolymphatic channels have been demonstrated in post‐mortem temporal bone specimens [16, 25], in vivo investigations of their role in endolymph homeostasis are lacking. Based on our current findings, it can be stated that the ES/ED complex seems to play a crucial role in the development of ELH, although the exact pathomechanism remains uncertain. Here, multi‐timepoint measurements visualizing the fluid dynamics (i.e., longitudinal flow, radial flow, dynamic flow, or no significant flow at all [26]) in the ES/ED complex could further illuminate the exact physiology. Additionally, higher resolution imaging approaches could aid in the exact localization of this CA uptake, i.e., revealing if these observations constitute “true” ES/ED signal intensity, or rather CA uptake in the aforementioned peri‐endolymphatic channels.

It should be noted that this is the first study that correlated radiographic ES/ED signal intensity markers with detailed, volumetric measurements of the endolymphatic space in a large cohort of both vestibular healthy controls and patients (with and without ELH). Our findings of an inverse relationship between ES/ED visibility (either as “true” ES/ED visibility, or as an in vivo surrogate imaging marker of peri‐endolymphatic channel CA uptake) and endolymphatic space, independent of clinical diagnosis or the presence of ELH, therefore imply a physiological relationship that might be modulated in MD. In general, this relationship, however, seems to be a sign of a basic, universal mechanism of inner ear fluid dynamics in both healthy and diseased inner ears.

Overall, these findings are in line with our previous findings of a symmetrical, low‐grade ELH in VM, and an asymmetrical, higher grade ELH in MD [7]. Additionally, in the current study, the clinical symptoms described by patients correlated well with ES/ED visibility, independent of diagnosis: while unilateral, asymmetrical cochlear symptoms like aural fullness or tinnitus (i.e., symptoms typical for MD) were associated with an ipsilaterally decreased ES/ED visibility, bilateral and symmetrical cochlear symptoms (i.e., symptoms more commonly observed in VM) were associated with a bilateral, symmetric high ES/ED visibility. When analyzing symptoms typical for VM (e.g., phono−/photophobia, headache), again, a bilateral, symmetric high ES/ED visibility was observable. Therefore, a clear link between clinical symptomatology and ES/ED‐visibility seems plausible. Based on these findings, a complete imaging‐based diagnostic workup of vestibular disorders would therefore ideally include both ELH assessment and ES/ED visibility rating (using delayed, post‐contrast MRI), paired with temporal bone morphology investigations (using high‐resolution CT) for clinical endotyping of e.g., MD subtypes. These different modalities require adequate, inner‐ear centered and geometry‐preserving image co‐registration [27]. As a simple “rule of thumb” classification, VM patients would typically present with (a) high bilateral ES/ED visibility, (b) no or low‐grade ELH, and (c) normal temporal bone morphology, whereas MD patients would exhibit (a) severe ELH on the affected side, and no or low‐grade ELH on the unaffected side, (b) low ES/ED visibility on the affected side, and medium ES/ED visibility on the contralateral side, and (c) ES/ED hypoplasticity or degeneration in temporal bone CT. Note that imaging alone cannot replace clinical reasoning, and should only be used to support clinical diagnoses.

The bilateral reduced ES/ED‐visibility in MD patients, compared to the HC and VM cohorts, fits well with earlier observations by Bächinger et al. who showed that ES/ED hypoplasia or degeneration correlates with bilateral disease progression in a long‐term MD cohort [28]. In a follow‐up study, the authors could demonstrate lower ES/ED signal intensity specifically in the degenerative endotypes of MD [29]. The ES/ED‐scale from our study could therefore provide a simple metric for the progression risk of MD to the contralateral ear in MD, which after 20 years may reach more than 40% [30, 31]. However, it should be noted that MD patients with a hypoplastic endotype [15] would exhibit inherently lower ES/ED visibility. Here, further research and longitudinal studies are necessary.

As a potential confounder of ELH volume analysis, the higher age of MD patients compared to VM patients needs to be considered. Earlier studies using volumetric analysis had shown an increase in endolymphatic volume in elderly healthy individuals [7, 32]. Other studies, which did not find a relationship between age and ELH, only employed semi‐quantitative grading [33]. Potentially, small age effects may go undetected without the use of advanced, three‐dimensional methods such as VOLT. Note that in the current analysis, age effects on ELH volume and ES/ED visibility were demonstrated independent of clinical diagnosis, i.e., also in healthy controls.

As a potential limitation, it should be noted that the ES/ED‐visibility scale was tested on 3D‐FLAIR images with the aforementioned sequence characteristics. Other sequences for ELH imaging exist, e.g., 3D inversion recovery (3D‐IR [34]), or the HYDROPS‐ [35] and HYDROPSMi2‐pipeline [36] which utilize two sets of 3D‐FLAIR images finetuned to perilymphatic and endolymphatic fluid intensity. Due to the different imaging characteristics of these sequences, the applicability of the ES/ED scale for other methods of ELH visualization is currently unclear. However, similar to how VOLT (which was initially developed on FLAIR‐images) could be shown to be applicable also in the HYDROPSMi2‐pipeline [4, 37], an extension of the ES/ED scale to other ELH imaging pipelines could be possible and should be investigated in future studies.

## 5 Conclusions

Assessment of ES/ED visibility in contrast‐enhanced, delayed inner ear MRI using a novel semi‐quantitative scale may support clinicians in the differentiation between episodic vertigo syndromes caused by MD or VM. Paired with endolymphatic hydrops imaging, ES/ED visibility assessment might additionally contribute to the understanding of the development and clinical course of inner ear disorders.

## Conflicts of Interest

The authors declare no conflicts of interest.
